# Common Habits, Adverse Events, and Opinions Regarding Pre-Workout Supplement Use Among Regular Consumers

**DOI:** 10.3390/nu11040855

**Published:** 2019-04-16

**Authors:** Andrew R. Jagim, Clayton L. Camic, Patrick S. Harty

**Affiliations:** 1 Sports Medicine, Mayo Clinic Health System, Onalaska, WI 54650, USA; 2 Kinesiology and Physical Education, Northern Illinois University, DeKalb, IL 60115, USA; ccamic1@niu.edu; 3 Exercise and Performance Nutrition Laboratory, Lindenwood University, St. Charles, MO 63301, USA; pharty@lindenwood.edu

**Keywords:** ergogenic aid, supplement, pre-workout, adverse events, caffeine, niacin, beta-alanine, performance, strength, power

## Abstract

The purpose of the present study was to examine characteristics of multi-ingredient pre-workout supplement (MIPS) users, their common patterns/habits of MIPS ingestion, and their associated feelings about the effectiveness and safety of this class of supplements. An online electronic survey was distributed through social media to assess self-reported supplementation practices and preferences among adult males and females who reported regular MIPS use. A total of 1045 individuals responded, with 872 of these individuals (males: *n* = 636, 72.9%; females: *n* = 233, 26.7%; mean ± SD; age = 27.7 ± 7.9 years; training age = 8.2 ± 7.3 years) completing the survey. The majority of respondents reported the length of current or past MIPS consumption as greater than one year (*n* = 630, 72.2%), with ingestion frequencies primarily of four (*n* = 210, 24.1%) or five (*n* = 212, 24.3%) days per week of training. In addition, the three most popular goals for ingesting MIPS were increased energy and focus (*n* = 776, 89.0%), muscular endurance (*n* = 325, 37.3%), and blood flow or “pump” (*n* = 322, 37.0%). Although most users reported ingesting one serving size with each use, 14% reported ingesting two or more, and 18% indicated they ingest MIPS more than once per day. Importantly, over half (54%) of the respondents reported experiencing side-effects following MIPS use, including skin reactions, heart abnormalities, and nausea. Females were more likely than males to experience these side effects, despite being less likely to consume two or more serving sizes per dose. Our findings also indicated that MIPS users should consume no more than the recommended serving size of a given supplement, as the potentially significant variability in the caffeine content of MIPS products is compounded as more doses are consumed. Furthermore, MIPS users should minimize the ingestion of other supplements which contain high levels of niacin and caffeine, as the concurrent consumption of such products may put users above the tolerable upper limits for these substances.

## 1. Introduction

The use of dietary supplements to enhance exercise performance is becoming an increasingly popular strategy, particularly among fitness enthusiasts and athletes. As of 2012, a report by Kantor et al. [1] indicated that approximately 52% of US adults reported using dietary supplements on a regular basis, which is similar to trends of use reported from the early 2000s [2]. There is reason to believe these prevalence rates may be even higher today, as economic sales from dietary supplements have continued to rise. In 2016, it was estimated that the overall economic impact of the dietary supplement industry was approximately $122 billion, with future projections estimating a potential worth of nearly $278 billion by the year 2024 [3]. With such an increase in dietary supplement use, it is important to examine the behaviors of supplement users and key motivators for use in order to better understand potential areas for concern and identify strategies to ensure safe and effective dietary supplement use.

A sub-set of dietary supplements that has garnered attention from regular exercisers are multi-ingredient pre-workout supplements. These products often contain a blend of ingredients purported to improve acute exercise performance following ingestion, which may augment training adaptations if ingested over a longer period when used in conjunction with a structured training program. A recent review by Harty and Zabriskie et al. [4] summarized the available literature regarding the efficacy and safety of MIPS use with regards to single ingestion and repeated use. There appears to be supporting evidence that MIPS supplementation can improve exercise performance following a single use, particularly in the context of muscular endurance-based activities [4,5,6,7,8,9,10,11,12]. Furthermore, extended use appears to enhance training adaptations with a low risk for adverse events [4,13,14,15,16,17,18,19], assuming consumers are following the manufacturer recommendations for proper use. 

To date, a paucity of information is available regarding the usage patterns of individuals who routinely ingest MIPS. Every MIPS product has a specific serving size recommendation for consumption. However, users may opt to consume less or more than the recommended serving size, which could present unknown benefits or risks. The usage patterns of MIPS users are relevant in the light of findings from a recent study that examined the common ingredient profiles of top-selling pre-workout supplements [20]. The authors of this study indicated three important findings regarding pre-workout supplement labels: a) Many ingredients are under-dosed when compared to evidence-based guidelines on recommended doses; b) many ingredient quantities are not provided; and c) some ingredients, such as niacin, may be over-dosed and potentially dangerous, particularly if consumers ingest other niacin containing compounds. Thus, highlighting consumers’ perceptions of their dosing regimens and feelings of safety is important. Therefore, the aim of the current study was to examine characteristics of MIPS users, their common patterns/habits of MIPS ingestion, and their associated feelings about the effectiveness and safety of this class of supplements.

## 2. Materials and Methods

This cohort study utilized an online electronic survey that was developed and electronically distributed via social media, to assess self-reported practices, preferences, and reasons for use among regular MIPS users over a span of 3 months. Males and females between the ages of 18 and 65 who reported regular MIPS use were recruited to participate in this online survey. 

### 2.1. Subjects

A total of 1045 individuals responded to the survey, with 872 of these individuals (males: *n* = 636, 72.9%; females: *n* = 233, 26.7%; mean ± SD age = 27.7 ± 7.9 years; training age = 8.2 ± 7.3 years) completing the survey. The primary mode of exercise for these subjects (*n* = 872) was resistance training (*n* = 731, 83.8%), aerobic training (*n* = 81, 9.3%), group exercise classes (*n* = 33, 3.8%), and recreational activities (*n* = 26, 3.0%), with training frequencies of one (*n* = 5, 0.6%), two (*n* = 15, 1.7%), three (*n* = 93, 10.7%), four (*n* = 234, 26.8%), five (*n* = 311, 35.7%), six (*n* = 180, 20.6%), or seven (*n* = 33, 3.8%) days per week. Participants were informed of the survey design and target outcomes and provided electronic consent before completing the survey. This survey was approved by the Institutional Review Board of Northern Illinois University in compliance with the Human Subjects Guidelines.

### 2.2. Survey

The online survey (Qualtrics, Provo, UT, USA) was developed by the authors and preliminarily distributed to other experts in the field who provided suggestions and feedback on how to improve the content validity and usability of the research tool. The survey consisted of 23 questions with the intention to identify basic demographic information, training history, frequency of MIPS use, number of serving sizes ingested, desired outcomes, preferred ingredients, and adverse events associated with MIPS use. There were three open-ended questions: (1) Age, (2) preferred brand of MIPS, and (3) years spent regularly training. The survey was distributed online via multiple social media platforms, targeting individuals who previously or were currently regularly consuming pre-workout supplements. The survey was also distributed electronically and emailed to several faculty members and then disseminated to students of exercise science programs throughout the United States. The survey was active from October–December 2018.

### 2.3. Statistical Analysis

The data were analyzed to present descriptives for all demographic data and closed-answer questions. These data are presented as means and standard deviation (SD). Chi square tests were used to compare outcomes for select closed-ended questions grouped by sex. A significance threshold of 0.05 was used for all analyses. All data were analyzed using SPSS V.25 (IBM Corporation, Armonk, NY, USA). 

## 3. Results

Subjects (*n* = 872) reported the length of current or past pre-workout supplement consumption as < 3 months (*n* = 65, 7.5%), 3–6 months (*n* = 77, 8.8%), 6–12 months (*n* = 99, 11.4%), or > 1 year (*n* = 630, 72.2%), with an ingestion frequency of one (*n* = 65, 7.5%), two (*n* = 90, 10.3%), three (*n* = 163, 18.7%), four (*n* = 210, 24.1%), five (*n* = 212, 24.3%), six (*n* = 89, 10.2%), or seven (*n* = 42, 4.8%) days per week of training. Relative to the pre-workout window, subjects reported ingesting their supplement ≤ 15 (*n* = 388, 44.5%), 30 (*n* = 413, 47.4%), 45 (*n* = 52, 6.0%), or ≥ 60 (*n* = 15, 1.7%) minutes prior to their workout. Table 1 provides the frequencies, percentages, and sex differences in responses to survey questions related to common practices of pre-workout ingestion, feelings of safety, and adverse events experienced.

Participants selected the following as primary goals when ingesting a pre-workout supplement: increased energy and focus (*n* = 776, 89.0%), muscular endurance (*n* = 325, 37.3%), blood flow or “pump” (*n* = 322, 37.0%), muscular strength (*n* = 260, 30.0%), increased muscle mass (*n* = 113, 13.0%), fat loss (*n* = 66, 7.6%), and other (*n* = 30, 3.4%). Table 2 highlights key ingredients that subjects looked for and the most important factors they considered when choosing a pre-workout supplement. Table 3 presents a list of the top 15 pre-workout supplement brands reported by the users in the present cohort.

## 4. Discussion

The aim of the current study was to better understand how regular MIPS users utilize these products, specifically regarding serving size use, frequency of use, primary reasons for use, and desired ingredients. A secondary aim was to examine their opinions about safety and the incidence of adverse effects. Of the individuals who completed the survey, the majority of the MIPS users were males who regularly participated in resistance training and exercised four to six times per week. The high prevalence of MIPS use by males in the present sample aligns with the results of a recent survey which showed that a higher proportion of male NCAA Division I athletes reported pre-workout supplement use compared to female athletes [21]. In addition, the majority of MIPS users (72%) in the current study reported consuming MIPS products for greater than one year with an average ingestion frequency of four to five times per week, which appears to coincide with self-reported training frequency. This is an important finding, as there is currently a paucity of long-term safety data available regarding pre-workout supplementation. As highlighted in the review by Harty et al. [4], short-term ingestion (less than eight weeks) does not appear to negatively influence markers of clinical health, but less is known regarding the long-term safety of these products. Therefore, long-term safety trials are certainly warranted, particularly as the majority of users in the current study reported regularly consuming MIPS for longer than one year. 

The majority of the MIPS users in the current study appeared to follow manufacturer guidelines, as the most common reported ingestion times were 15–30 min prior to exercise, as is recommended on most supplement labels. Although most users reported ingesting one serving size with each use, 14% reported ingesting two or more, and 18% indicated they ingest MIPS more than once per day. Males were more likely than females to follow this supplementation pattern. Currently, it is unknown whether a dose-response relationship exists between the number of serving sizes ingested and performance outcomes. A recent study sought to examine the effects of one or two servings of a pre-workout supplement on fuel utilization and perceptions of fatigue in recreational runners (in review), with preliminary evidence indicating that two servings of a MIPS appeared to lower rating of perceived exertion (RPE) during a 30 min treadmill run, while also resulting in significantly higher systolic and diastolic blood pressure, with no changes in hemodynamic variables observed following ingestion of a single serving. As discussed earlier regarding the lack of safety evidence with MIPS use, even less information is available regarding the long-term safety of ingesting double the recommended serving size. Therefore, consumers may want to exercise caution prior to engaging in this practice, particularly if other dietary supplements are being consumed concurrently. For example, nearly a third of MIPS users reported combining a MIPS with other caffeine containing products, and if more than one serving size is also ingested, one could approach dangerous levels of caffeine consumption, as MIPS tend to contain 250–400 mg of caffeine per serving [20,22]. Even more concerning is that several investigations have also demonstrated that the caffeine content in selected MIPS products varies by as much as 266% between batches, which further increases the chance for the consumer to consume excessive amounts of caffeine [22,23]. Additionally, some MIPS products tend to contain high amounts of niacin (vitamin B3) [20] and individuals who consume more than one serving at once or throughout the day in addition to other niacin-containing products could rapidly approach the tolerable upper limit of 35 mg/day [24]. These concerns surrounding excessive niacin consumption have recently been addressed in our manuscript detailing common ingredient profiles of MIPS products [20].

Slightly over half of the MIPS users in the current study reported that they had experienced side effects following MIPS use, with the most commonly reported side effects including skin reactions, heart abnormalities (self-diagnosed), and nausea. This high incidence of adverse events is potentially concerning, as most MIPS users reported that they followed the manufacturer guidelines on serving size. Our findings also indicated the females were more likely than males to experience these side effects, despite being less likely to consume two or more serving sizes per dose. It is difficult to discern the primary cause of these side effects, as the majority of MIPS contain proprietary blends of ingredients with varying amounts of ingredients and some ingredient amounts not disclosed [20]. However, the skin reactions are likely a result of the beta-alanine and niacin contained in MIPS products, which commonly result in paresthesia and a facial flushing effect, respectively. Despite the high incidence of side effects, 84% of users still believed MIPS ingestion to be safe and 61% would recommend a MIPS to others. Additionally, although not assessed in the current study, the time of day when MIPS are ingested may influence the likelihood of side effects or even sleep disturbances and subsequent degrees of recovery. Therefore, future research should assess whether the timing of MIPS ingestion influences adverse effects or negatively influences sleep.

The majority of MIPS users in the current sample reported increased energy and focus as a primary goal of ingesting MIPS products, with improved muscular strength, muscular endurance, and enhanced blood flow or a “pump” effect as other common outcomes. As a result, supplement users selected ingredients such as caffeine, beta-alanine, creatine, and vasodilators as the top ingredients that influenced product selection. Unsurprisingly, these ingredients align with the results of a recent analysis of the ingredient profiles of top-selling pre-workout products, which identified that beta-alanine, caffeine, citrulline, creatine, and arginine were among the most common ingredients found in top-selling MIPS products [20]. Interestingly, a small percentage of MIPS users reported that banned stimulants and anabolic agents were also a desired ingredient when selecting MIPS products. This preference has been reported previously, as a recent survey of German fitness studio visitors [25] found that 20% of the pre-workout users listed 1,3-dimethylamylamine (DMAA), a synthetic stimulant that is banned in the United States, as a desired ingredient for its stimulatory benefits. Improving performance tends to be a common reason for dietary supplement use among regular exercisers and athletes, with correcting nutritional deficiencies and health benefits as secondary outcomes [26,27,28]. Although there appears to be a significant misunderstanding regarding rationale for supplement use and intended benefits among supplement users [29], it is important to note that all dietary supplements are required to have the following message included on the supplement label: “These statements have not been evaluated by the Food and Drug Administration. This product is not intended to diagnose, treat, cure, or prevent any disease.” Furthermore, supplement manufacturers often include specific instructions relating to serving size, directions for use and any pertinent warnings on how to store the product, how to increase or decrease serving size depending on tolerability, or warnings against use by certain special populations.

As a result, and in conjunction with the findings of our previous publication on the common ingredient profiles of pre-workout supplements [20], we recommend that consumers seek out educational resources on these products and closely examine: (1) Ingredient profiles; (2) manufacturer recommendations on serving size; (3) rationale for purported benefits; (4) concurrent supplement use; and (5) quality of brand when selecting a MIPS and how they are used. Thus, when determining whether to take a given pre-workout supplement, consumers and healthcare professionals should prioritize products that do not contain proprietary blends with undisclosed ingredients or amounts. This would help consumers understand exactly what they are ingesting and allow them to identify any ingredients that may be causing adverse reactions. Additionally, consumers should carefully examine the ingredient profile of the product to ensure that proven ergogenic ingredients, such as caffeine, creatine, and beta-alanine, are present in sufficient amounts [20]. Similarly, MIPS users should consume no more than the recommended serving size of a given supplement, as the potentially significant variability in the caffeine content of MIPS products is compounded as more doses are consumed. Furthermore, MIPS users should minimize the ingestion of other supplements that contain high levels of niacin and caffeine, as the concurrent consumption of such products may put users above the tolerable upper limits for these substances. Finally, to minimize the risk of ingestion of banned substances, we recommend that MIPS consumers use products from trusted brands that are certified by third-party organizations, such as Informed Choice, NSF, and the Banned Substances Control Group. 

### Limitations

As with any online survey research, this study has a number of potential limitations that may have influenced the validity of the data and sampling [30]. In particular, our survey had a relatively narrow targeted focus on current or previous MIPS users and was primarily distributed to college-aged individuals in the Midwest region of the United States. Thus, it is possible that the sampling bias of the survey respondents and their behaviors regarding MIPS use may not be reflective of the overall target population. Wright [30] also addresses other disadvantages and limitations with online survey research including: (1) lower incentive for respondents to provide honest answers with no interviewer present, (2) potential for different interpretations of survey answers, (3) lower validity rates with closed-end questions, and (4) relying on the accuracy of respondents’ memory. Although these issues were unavoidable in the present study, the findings of our survey should be considered with these factors in mind. 

## 5. Conclusions

This investigation identified common usage habits of multi-ingredient pre-workout supplement users, provided novel demographic data for this population, and reported the perceptions of MIPS users regarding the safety and efficacy of this common class of dietary supplement. The results of this survey suggest that many consumers have used MIPS products multiple times per week for more than one year. In addition, many respondents reported ingesting more than one serving of MIPS per use, consuming doses larger or smaller than manufacturer recommended serving sizes, or consuming MIPS products more than once per day. Alarmingly, more than one third of respondents reported that they consumed MIPS along with other caffeine-containing products. Furthermore, more than half of the respondents to this survey reported that they had experienced deleterious side effects associated with MIPS use. In light of these results, it is evident that future studies must be conducted to determine the safety of long-term (greater than one year) MIPS supplementation as well as the safety of multiple doses of MIPS products consumed at one time. 

## Figures and Tables

**Table 1:** Common practices of pre-workout supplement users.

Survey Question	Answers	All	Males	Females	*χ* ^2^	*p* Value
When ingesting a pre-workout supplement, how many serving sizes do you typically ingest?	One	746 (85.6%)	533 (83.9%)	212 (91.0%)	7.815	0.050
	Two	118 (13.5%)	97 (15.3%)	20 (8.6%)		
	Three	3 (0.3%)	2 (0.3%)	1 (0.4%)		
	Four	3 (0.3%)	3 (0.5%)	0 (0.0%)		
Do you follow the recommended serving size or dosing instructions on the pre-workout label?	Yes	553 (63.4%)	396 (62.4%)	157 (67.4%)	1.886	0.389
	Sometimes	257 (29.5%)	194 (30.6%)	61 (26.2%)		
	No	60 (6.9%)	45 (7.1%)	15 (6.4%)		
Do you ingest pre-workout on non-training days?	Yes	39 (4.5%)	29 (4.6%)	10 (4.3%)	0.056	0.972
	Sometimes	120 (13.8%)	87 (13.7%)	33 (14.2%)		
	No	711 (81.5%)	519 (81.7%)	190 (81.5%)		
Do you ever ingest a pre-workout supplement more than once during a single day?	Yes	156 (17.9%)	127 (20.0%)	29 (12.4%)	6.597	0.010
	No	714 (81.9%)	508 (80.0%)	204 (87.6%)		
Do you ever ingest your pre-workout supplement with other caffeine-containing products?	Yes, but not always	265 (30.4%)	190 (30.0%)	75 (32.3%)	0.630	0.730
	Yes, all the time	39 (4.5%)	30 (4.7%)	9 (3.9%)		
	No	563 (64.6%)	413 (65.2%)	148 (63.8%)		
Do you feel as though consuming a pre-workout supplement in general is safe?	Yes	764 (87.6%)	561 (88.5%)	202 (87.4%)	0.176	0.675
	No	103 (11.8%)	73 (11.5%)	29 (12.6%)		
Do you feel as though consuming a pre-workout supplement, at the dose you regularly consume, is safe?	Yes	740 (84.9%)	548 (86.4%)	192 (82.8%)	6.291	0.043
	No, so I don’t on a regular basis	52 (6.0%)	29 (4.6%)	21 (9.1%)		
	No, but I continue to do it anyway	76 (8.7%)	57 (9.0%)	19 (8.2%)		
Do you feel as though consuming a pre-workout supplement improves your workout?	Definitely yes	474 (54.5%)	353 (55.7%)	121 (52.2%)	4.302	0.367
	Probably yes	264 (30.3%)	194 (30.6%)	69 (29.7%)		
	Might or might not	112 (12.8%)	76 (12.0%)	35 (15.1%)		
	Probably not	13 (1.5%)	7 (1.1%)	6 (2.6%)		
	Definitely not	5 (0.6%)	4 (0.6%)	1 (0.4%)		
Would you recommend, or have you recommended to others, that they should consume a pre-workout supplement before exercise?	Yes	532 (61.0%)	394 (62.2%)	137 (59.3%)	3.175	0.204
	Maybe	203 (23.3%)	152 (24.0%)	51 (22.1%)		
	No	131 (15.0%)	87 (13.7%)	43 (18.6%)		
Have you ever experienced any side-effects or adverse events following pre-workout supplement ingestion?	Yes	471 (54.0%)	328 (51.8%)	141 (61.0%)	5.8	0.016
	No	395 (45.3%)	305 (48.2%)	90 (39.0%)		
If you have experienced any adverse events following pre-workout ingestion, which of the following occurred?	Light-headedness	167 (19.2%)	126	39		
	Dizziness	128 (14.7%)	95	32		
	Nausea	223 (25.6%)	158	64		
	Heart abnormalities (e.g., rapid heart rate, palpitations)	204 (23.4%)	147	55		
	Skin reactions (e.g., flushing, rash, irritation, itchiness)	299 (34.3%)	205	93		
	Other	116 (13.3%)	83	32		

**Table 2:** Factors that influenced pre-workout supplement selection.

**Key Ingredients**	***n***	**%**
Caffeine	700	80.3
Beta-alanine	505	57.9
Creatine	402	46.1
Vasodilators	380	43.6
Branched-chain amino acids	30	3.4
Vitamins	62	7.1
Banned stimulants	50	5.7
Banned anabolic agents	30	3.4
Other	62	7.1
**Factors Influencing Product Selection**	***n***	**%**
Included ingredients	583	66.9
Quality of product/independent verification	458	52.5
Price	408	46.8
Supported benefits from scientific publications	360	41.3
Reputable brand	347	39.8
Referral from trusted source	255	29.2

**Table 3:** Top MIPS brands listed (Total valid responses: *n* = 745).

Brand	Frequency
Cellucor	132
Beyond Raw by GNC	57
Optimum Nutrition	48
Jym Supplement Science	39
ProSupps	26
MusclePharm	22
1st Phorm	17
BSN	17
VPX	16
Redcon1	13
Advocare	13
Ghost	13
PEScience	13
GAT Sport	11
MyProtein	10
